# Interaction of Halictine-Related Antimicrobial Peptides with Membrane Models

**DOI:** 10.3390/ijms20030631

**Published:** 2019-02-01

**Authors:** Markéta Pazderková, Petr Maloň, Vlastimil Zíma, Kateřina Hofbauerová, Vladimír Kopecký, Eva Kočišová, Tomáš Pazderka, Václav Čeřovský, Lucie Bednárová

**Affiliations:** 1 Institute of Physics, Faculty of Mathematics and Physics, Charles University, Ke Karlovu 5, 121 16 Prague 2, Czech Republic; pazderkova@karlov.mff.cuni.cz (M.P.); malonp@karlov.mff.cuni.cz (P.M.); vlastimil.zima@gmail.com (V.Z.); hofbauer@karlov.mff.cuni.cz (K.H.); kopecky@karlov.mff.cuni.cz (V.K.J.); kocisova@karlov.mff.cuni.cz (E.K.); tomas.pazderka@matfyz.cz (T.P.); 2 Institute of Organic Chemistry and Biochemistry, v.v.i., Academy of Sciences of the Czech Republic, Flemingovo náměstí 2, 166 10 Prague 6, Czech Republic; cerovsky@uochb.cas.cz

**Keywords:** antibacterial peptides, halictine, circular dichroism, fluorescence, infrared spectroscopy

## Abstract

We have investigated structural changes of peptides related to antimicrobial peptide Halictine-1 (HAL-1) induced by interaction with various membrane-mimicking models with the aim to identify a mechanism of the peptide mode of action and to find a correlation between changes of primary/secondary structure and biological activity. Modifications in the HAL-1 amino acid sequence at particular positions, causing an increase of amphipathicity (Arg/Lys exchange), restricted mobility (insertion of Pro) and consequent changes in antimicrobial and hemolytic activity, led to different behavior towards model membranes. Secondary structure changes induced by peptide-membrane interaction were studied by circular dichroism, infrared spectroscopy, and fluorescence spectroscopy. The experimental results were complemented by molecular dynamics calculations. An α-helical structure has been found to be necessary but not completely sufficient for the HAL-1 peptides antimicrobial action. The role of alternative conformations (such as β-sheet, PPII or 3_10_-helix) also seems to be important. A mechanism of the peptide mode of action probably involves formation of peptide assemblies (possibly membrane pores), which disrupt bacterial membrane and, consequently, allow membrane penetration.

## 1. Introduction

Antimicrobial peptides (AMPs) are important participants in the initial response of immune systems and have been found in nearly all living organisms including bacteria, fungi, plants and animals [1,2,3,4]. Potentially, they offer alternatives to disease treatment as a replacement for common antibiotics, without disadvantages like resistance, allergies, etc. Many AMPs have been isolated and subsequently synthesized together with their analogs. Their antibacterial activities have been determined against Gram-negative and Gram-positive bacteria as well as against cancer cells [1,5,6,7,8]. A general lack of new antibiotics for the treatment of Gram-negative infections and a continuous increase in multi-drug resistance has recently caused a wave of interest in possible mechanisms of AMP action. One of the recognized effects is their ability to disrupt bacterial membranes *via* non-specific electrostatic interactions with the membrane lipid components [1]. There are two recognized common and important criteria for functionally active AMPs. These include a network of cationic charges and the ability to adopt an amphipathic structure, where hydrophobic and hydrophilic parts form oppositely oriented domains upon interaction with negatively charged bacterial membranes. The possible mechanisms of AMP action fall into two basic categories: (1) formation of pores in bacterial membranes *via* transmembrane penetration (e.g., the barrel stave and toroidal pore models) or (2) disruption of membranes (e.g., the carpet and detergent models) [1,8,9,10,11]. These then lead to the breakdown of the transmembrane potential causing leakage of the cell contents and finally cell death. The physico-chemical concept of such antibacterial action has been discussed and particular attention has been paid to changes of the phase state of the membrane [12,13,14].

Quite importantly, AMPs exhibit high preference for bacterial over mammalian cells. This is probably associated with known significant differences between mammalian and bacterial cell membranes [5,6,12,15,16]. The type of mammalian cell membrane is represented by the plasma membrane of red blood cells. This membrane consists of about 60% phospholipids and 25% cholesterol. Distribution of phospholipids between outer and inner lipid leaflets of the bilayer is asymmetric with neutral phospholipids phosphatidylcholine and sphingomyelin exposed to the extracellular matrix. On the other hand, negatively charged lipids such as phosphatidylglycerol, diphosphatidylglycerol or cardiolipin and the zwitterionic phosphatidylethanolamine are the main constituents of the cytoplasmic membrane of both Gram-positive and Gram-negative bacteria (having an additional layer of peptidoglycan and an outer membrane layer composed mainly of lipopolysaccharides). In a simplified way, the AMPs are exposed to a neutral membrane surface in the case of mammalian cells and to a negatively charged surface in the case of bacteria.

Within the last decade, several original discoveries of AMPs isolated from the venom of *Hymenoptera* insects have been made and described by our collaborators [17,18,19,20,21,22]. Biological activities of these new AMPs have been determined and compared to the activities of their synthesized analogs to consider their eventual pharmacological application. Based on initial electronic circular dichroism (ECD) investigations, the peptides may undergo substantial structural changes in the presence of simple membrane-mimicking models such as 2,2,2-trifluoroethanol (TFE) and sodium dodecyl sulfate (SDS). Moreover, peptide structural behavior can be substantially affected by primary structure modifications. In our initial study of peptides related to Halictine-1 (HAL-1), a short linear AMP containing 12 amino acids isolated from the venom of the eusocial bee *Halictus sexcinctus*, we demonstrated that HAL-1 and its analogs are able to form amphipathic structures when in α-helical conformation [19]. A subsequent detailed spectroscopic study of the natural HAL-1 [23] resulted in a nontrivial picture involving not only a significant role of α-helical conformation but also an important role of other arrangements including random coil, β-structure and/or polyproline II (PPII) structures. Overall, the results presented overwhelming complexity and implied a need for additional, more detailed studies. Here, we focus in detail on physico-chemical properties and structure-activity relations of peptides related to HAL-1 including their geometries, conformation and dynamic behavior in various situations like in solutions, or in interaction with different membrane models. Conformational changes have been induced by an interaction with (a) TFE—an α-helix forming solvent [24], (b) SDS micelles—a very simple bacterial membrane model [25,26] and also by (c) liposomes of different phospholipid composition presented by a combination of various concentration mixtures of 1,2-dimyristoyl-*sn*-glycerol-3-phosphatidylcholine (PC) and 1,2-dimyristoyl-*sn*-glycero-3-phospho-(1′-*rac*-glycerol) (PG)—systems more accurately mimicking mammalian and bacterial membranes [25,26].

Inspired by the already presented analogues with specific sequence modifications and their known biological activities [19], here, we present a study of HAL-1 analogs (Table 1) with possible therapeutic potential (i.e., the analogs exhibiting potent activities against various pathogens while having substantially reduced hemolytic activity against red blood cells). Particularly, HAL-1/10 and HAL-1/20 analogs look promising for potential therapeutic applications because these peptides lack undesired hemolytic activities, while their antibacterial potencies, especially against *P. aeruginosa*, are higher than for natural HAL-1. A combined use of infrared (IR), circular dichroism (electronic (ECD) and vibrational (VCD)) and fluorescence spectroscopies allows us to obtain complex information about structural changes of the peptides upon interaction with model membranes. The set of IR, ECD, and fluorescence spectroscopy experiments performed at room temperature is complemented by time- and temperature-dependent ECD measurements, which allows us to distinguish and describe even subtle conformational changes of the peptides in interaction with membrane-mimicking environments, and by ECD and VCD study of a concentration dependency of HAL-1 analogs. Utilization of these experimental methods might help us to better understand the relation between the peptide primary/secondary structure changes and elucidate the mechanisms of the HAL-1 peptides action. A correlation between the peptide structural changes and biological activities can be also determined. The experimental data are compared to molecular dynamics (MD) simulations of HAL-1 in interaction with model membranes.

## 2. Results and Discussion

In our study, we investigate effects of changes in ionicity, hydrophobicity, flexibility and/or amphipathicity of the chosen peptides induced by an exchange of selected residues by Lys and Pro residues (see Table 1) on their structural behavior in membrane-mimicking environments represented by TFE, SDS micelles and phosphatidylcholine/phosphatidylglycerol-based liposomes. Based on the simple peptide structural prediction [28], the substitution of amino acids Ser4 and His9 (HAL-1/10), Met2 and His9 (HAL-1/20) or Arg12 (HAL-1/6) by Lys stabilizes the α-helical conformation, and the replacement of two amino acids by Lys (HAL-1/10 and HAL-1/20) improves the helical amphipathicity of the peptides (see Table 1). On the contrary, insertion of Pro9 (HAL-1/2) may cause structural irregularity, as Pro often breaks regular structures.

### 2.1. Structural Changes Followed by ECD

#### 2.1.1. Structural Changes Due to the Presence of TFE and SDS 

**Figure 1:**
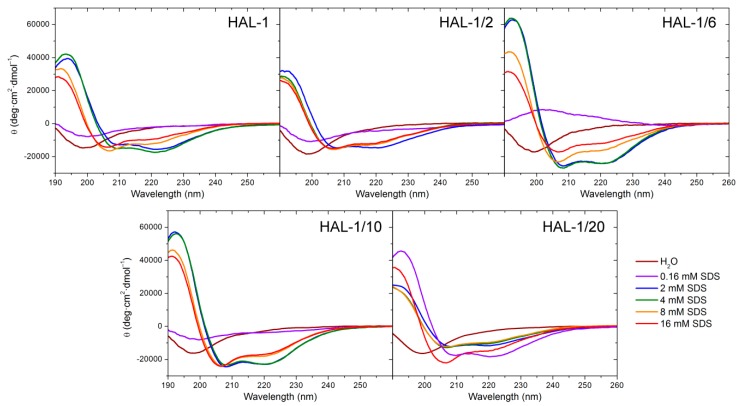
ECD spectra of HAL-1 and its analogs (0.125 mg/mL) in aqueous solution and in the presence of SDS (0.16, 2, 4, 8, and 16 mM).

Native HAL-1 in the aqueous environment, as well as all HAL-1 analogs, show a predominantly unordered structure, characterized by a negative ECD band at ~198 nm [29] (Figure 1). Upon addition of TFE, ECD spectra undergo a shape change. Formation of double negative minima at ~205 and 222 nm indicates a gradual appearance of an α-helical component (an isodichroic point at 202 nm suggests a two-state conformational change) (not shown). According to the two-state model [30] (Table A1), the presence of 30% TFE (*v*/*v*) causes a ~20−30% increase in the α-helical content, depending slightly on the particular primary structure. In the presence of SDS, the spectral changes appear more complex (Figure 1). The process conditioned by SDS is contributed not only by unordered and α-helical conformations, but also by secondary structures like a β-sheet or PPII helix [31]. Interaction with various proportions of SDS occurs in several stages, and a simple process of the two-state equilibrium does not describe sufficiently all the observed structural changes (Table A1 and Table A2). At low concentrations (less than 2 mM, i.e., below critical micelle concentration (cmc)) SDS causes an intensity decrease of ECD curves. In the case of HAL-1/6 (Arg12 replaced by Lys), this process causes even a sign flip. At low concentrations SDS does not act as a membrane model but serves as a denaturation agent [32], thus the ECD curves under such conditions offer two possible explanations: either the original unordered/PPII structure adopts a conformation with a higher β-sheet content, or it becomes a truly statistical random conformation due to an interaction with SDS molecules. These two structures can hardly be distinguished on the basis of ECD. Rather different spectral behavior is observed for the analog HAL-1/20, for which the formation of a positive band at 194 nm and negative bands at 208 and 222 nm typical for α-helical conformation is observed even for 0.16 mM SDS concentration (i.e., far below cmc). Moreover, the ECD bands at 194 and 222 nm exhibit, under these conditions, the highest spectral intensities. Formation of the α-helical conformation for HAL-1/20 below cmc could be due to the substitution of Met2 by Lys which favors interaction with anionic SDS molecules and increases the peptide polarity and amphipathicity (see Table 1) [27]. For all the analogs except for HAL-1/20, with the increase of SDS concentration above cmc (i.e., when SDS starts acting as a crude membrane model) [33], we observe a pronounced increase of the α-helical content (formation of negative maxima at 208 and 222 nm and an increase of overall spectral intensity; Figure 1). For these peptides, additional spectral changes appear with a further increase of SDS concentration above 8 mM. At first the negative maximum at 208 nm shifts to 205 nm with the preserved band intensity, and the intensity of the negative maximum at 222 nm discernibly decreases. Then, an overall ECD intensity decreases and the maxima at 205 nm and 222 nm shift to 206 nm and 219 nm, respectively. This could be due to an additional formation of 3_10_-helix, PPII or β-structure [34]. For HAL-1/20 such spectral changes are observed for SDS concentration above 4 mM SDS.

The numerical ECD analysis confirms these qualitative findings (Table A1). As for HAL-1, for HAL-1/6 and HAL-1/10 the maximal α-helical content is achieved in 2 mM SDS while for HAL-1/20, it is maximal in 0.16 mM SDS. For SDS concentration above 4 mM, numerical analysis indicates a slight decrease of the α-helical and unordered structure content and a subtle increase of β-structures (β-sheet and β-turn) for all the peptides except for HAL-1/20. For 16 mM SDS, the α-helical structure still dominates at the expense of other structures (Table A2). As expected, the substitution of His9 by Pro in HAL-1/2 decreases the ability of the peptide to form an α-helix (the α-helical fraction does not exceed 50%). However, the numerical ECD data analysis, even with included PPII and the 3_10_-helical structure, can provide only a rough estimation of observed spectral changes depending on the available reference set [35,36,37,38].

**Figure 2:**
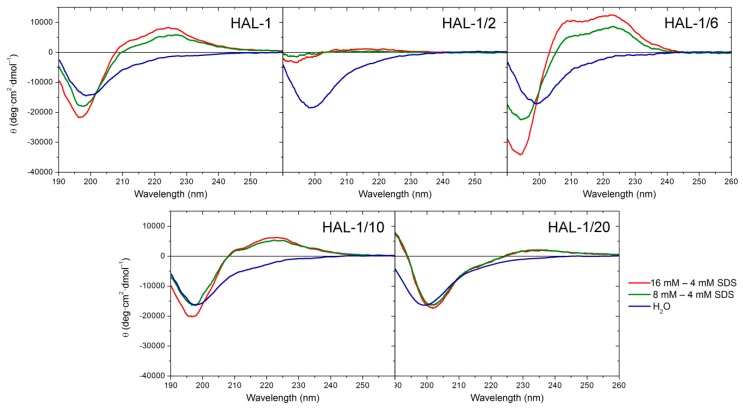
Difference of ECD spectra of HAL-1 and its analogs (0.125 mg/mL) in 8 mM SDS and in 16 mM SDS. ECD spectrum of the sample with the highest α-helical content in SDS solution (4 mM SDS peptide solution for HAL-1/2, HAL-1/6, HAL-1/10, and 0.16 mM SDS peptide solution for HAL-1/20 is taken as a reference). ECD spectra of the peptides in aqueous solution are depicted for comparison.

**Figure 3:**
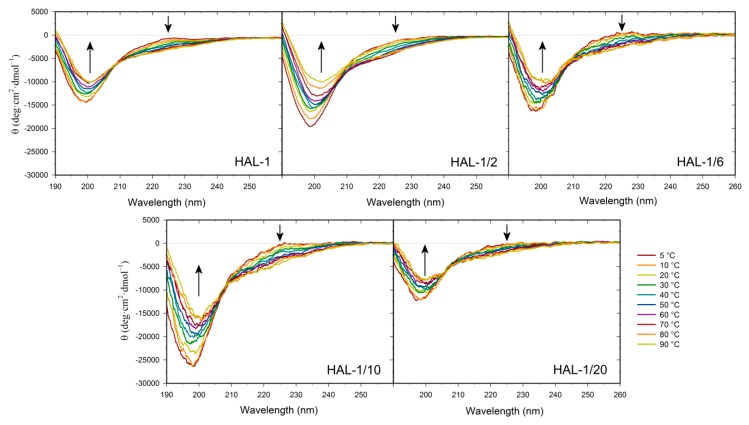
Thermal dependence of ECD spectra of HAL-1 and its analogs (0.125 mg/mL) in aqueous solution. The arrows show the direction of spectral changes with temperature increase from 5 to 90 °C (with a step of 10 °C).

**Figure A1:**
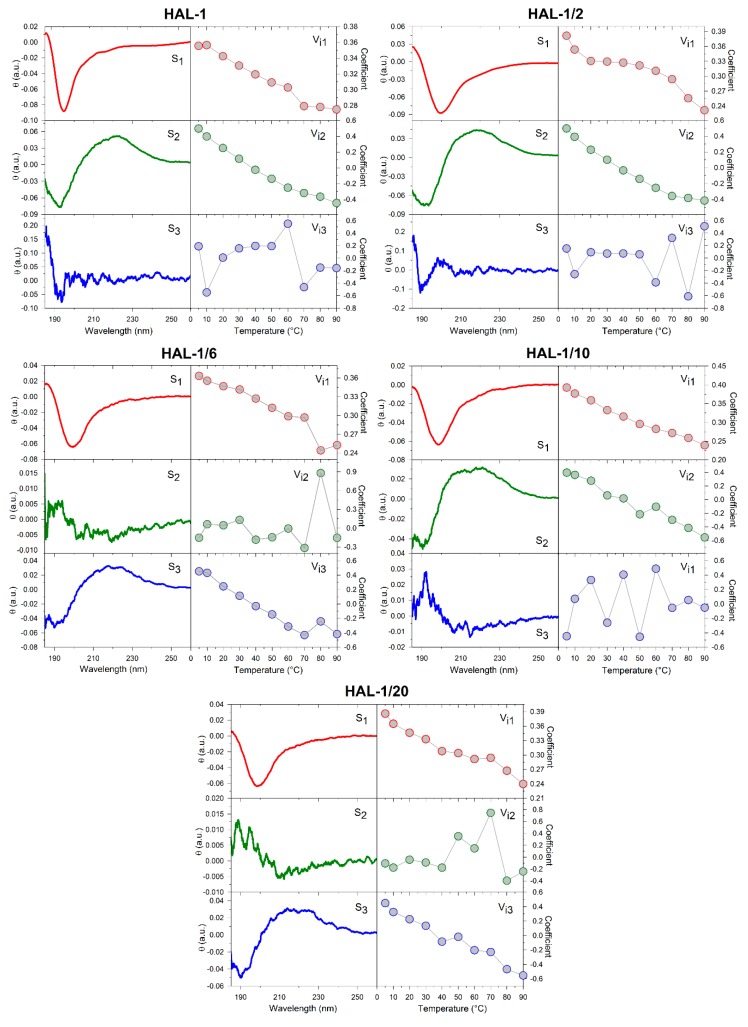
PCA results applied to the set of thermal dependence of ECD spectra (from 5 to 90 °C) of HAL-1 and its analogs. *S*_j_ represents the three most significant subspectra, and *V*_ij_ represents the appropriate coefficients indicating the relative contribution of the corresponding subspectrum.

We have previously suggested that, for natural HAL-1, additional spectral changes in the presence of SDS could originate in an alternation of the PPII structure content [23]. In order to recognize the PPII structure in ECD spectra of HAL-1 analogs in SDS solution, we have combined differential ECD spectra with ECD spectra of the thermal denaturation (Figure 2 and Figure 3) [31,39,40]. For all the studied peptides, differential ECD spectra indicate that a temporary increase of the α-helical content is followed by an additional structural reorganization—probably either a PPII structure formation or a decrease of a β-sheet content characterized by a positive band at ~225 nm, whose intensity increases with increasing SDS concentration (Figure 2). HAL-1/2 undergoes these changes only moderately, probably due to the presence of Pro residue, which may cause conformational stiffness. The temperature-dependent ECD spectra of HAL-1 analogs exhibit similar features. At low temperature (5 °C) they show the negative band at ~199 nm and the weaker positive band at ~220 nm (Figure 3). With a temperature increase, both of these bands decrease in intensity. An isodichroic point at ~210 nm indicates a two-state transition with decreasing PPII structure content [31,39]. At low temperature (5 °C), distinct spectral intensities of the analogs’ ECD curves indicate differences in peptide structural arrangement. While HAL-1/10 seems to have the highest portion of PPII structure and higher flexibility, HAL-1/20 appears to possess the highest fraction of unordered structure and less flexibility in its arrangement. Principal component analysis (PCA) indicates that thermal denaturation of the peptides probably leads to an increase of the β-structure content at the expense of decreasing percentage of unordered and/or PPII conformations [39] (Figure A1). Higher temperature seems to have similar effects on the peptides’ secondary structure if SDS acts as a denaturant (i.e., below cmc). The observed trends support the assumption that PPII conformation allows the polypeptide chain to switch easily to an α-helical or β-sheet and β-turn conformation [31,39].

#### 2.1.2. Structural Changes Due to the Presence of LUVs

**Figure 4:**
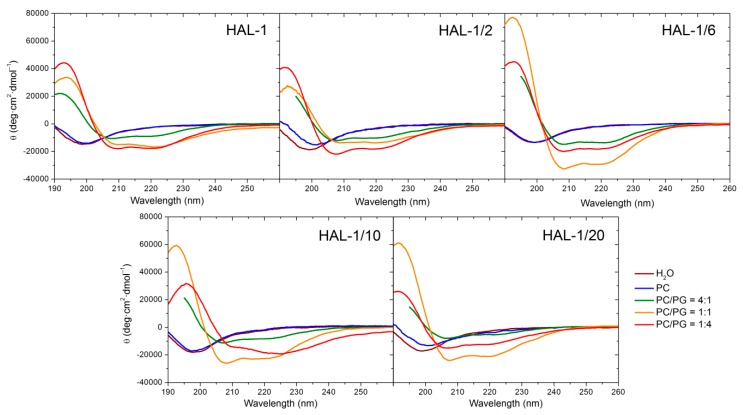
ECD spectra of HAL-1 and its analogs (0.125 mg/mL) in aqueous solution and in the presence of LUVs (L/P = 20) prepared from PC and PC/PG mixtures: 4:1, 1:1 and 1:4.

**Figure 5:**
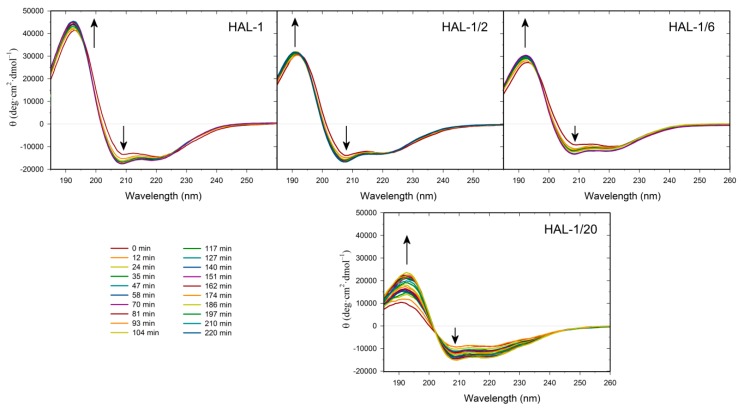
Time dependence of ECD spectra of HAL-1 and its analogs (0.125 mg/mL) in PC/PG = 1:4 mixtures (L/P = 20), measured within a 280-min time interval after the sample preparation with a 12-min step. The arrows show the direction of spectral changes with time.

**Figure A2:**
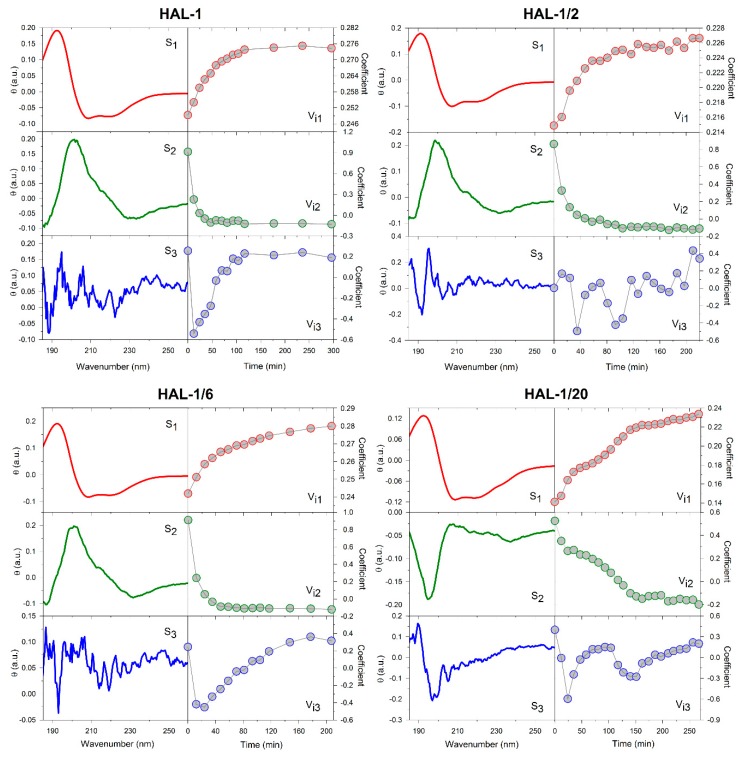
PCA results applied to the set of time dependence of ECD spectra of HAL-1 and its analogs in PC/PG = 1:4 mixtures measured within a 220 min time interval after the sample preparation with a 12-min time step. *S*_j_ represents the three most significant subspectra and *V*_ij_ represents the appropriate coefficients indicating the relative contribution of the corresponding subspectrum.

Neutral large unilamellar vesicles (LUVs) composed of PC were used as simple models of mammalian membranes. Similar to the natural HAL-1 [23], only subtle structural changes are observed in the ECD spectra of all the HAL-1 analogs upon addition of PC-based LUVs (Figure 4, Table A3). Slight conformational changes observed for HAL-1/2 and HAL-1/20 (Figure 4) likely correspond to an increase of β-sheet proportion. For HAL-1 and in part also for HAL-1/6, an α-helical structure can be induced when very high lipid/peptide (L/P) ratios (~600) are used (not shown). However, under such conditions, thorough analysis of ECD data is rather difficult due the limitations of ECD experiments (at high lipid concentrations, ECD spectra may be obscured due to light scattering on liposome molecules). The ability of all HAL-1 peptides to form the α-helical structure is enhanced in the presence of negatively charged PG-containing LUVs, representing a simple bacterial membrane model. Similar to HAL-1, for the proline-containing analog HAL-1/2, this enhancement is maximized with liposomes containing the highest fraction of PG (PC/PG = 1:4). For the other analogs, the maximal α-helical content is observed for the liposomes having the same fraction of PC and PG (PC/PG = 1:1) while an additional formation of β-structure occurs in the presence of the liposomes with the highest fraction of PG (PC/PG = 1:4) (Table A3). In order to obtain additional information about the structural stability of the peptides in the presence of PC/PG liposomes (composed of PC/PG in the 1:4 ratio), a time dependence of ECD spectra in the 280-min time interval has been studied for HAL-1 and its analogs HAL-1/2, HAL-1/6 and HAL-1/20 (Figure 5). The most pronounced spectral changes with time have been observed for HAL-1/20. This peptide shows the highest tendency to form a β-structure immediately upon interaction with PC/PG liposomes. A portion of the α-helical structure increases with time to a similar degree as for HAL-1/2. Following the PCA results (Figure A2), this structural change is compensated by a continuous β-structure content decrease. The structural behavior of HAL-1 seems to be comparable to HAL-1/6, with relatively small structural changes represented by a slight increase of the α-helical content. As expected, only minor structural changes are observed for HAL-1/2 (Figure 5).

Since the liposomes composed of PC/PG in various ratios roughly simulate bacterial and mammalian membranes, an attempt can be made to correlate the secondary structure changes inferred from the experimental ECD spectra with the peptide biological properties. The observed very limited interactions of HAL-1 analogs with the mammalian membrane (PC-based) models seem to correlate with their low (or none) hemolytic activities. Rather hemolytic analogs HAL-1 and HAL-1/6 show some reduced tendency to form the α-helical structure but such conformational change is induced only by a significant increase in lipid concentration. This is probably caused by the fact that the peptide activity against mammalian cells is much lower (~10–100×) than against bacterial cells, and the peptide propensity to interact with mammalian membrane model is therefore notably reduced. Behavior of the HAL-1 peptides towards PG-containing LUVs is very different. According to our data, all the HAL-1 analogs show a tendency to become α-helical upon this interaction. As the highest α-helical fraction is observed for HAL-1/6, which is less active than the native HAL-1, it seems that biological activities of the peptides are not solely determined by their propensity for forming an α-helical structure. The spectral changes observed for the HAL-1 analogs in interaction with liposomes of various PC/PG compositions indicate that, similar to the peptide behavior in the presence of SDS micelles (see above), an additional formation of β-structure and/or PPII conformation cannot be excluded and could be also important for their biological activities.

### 2.2. Structural Changes Followed by Infrared Spectroscopy 

**Figure 6:**
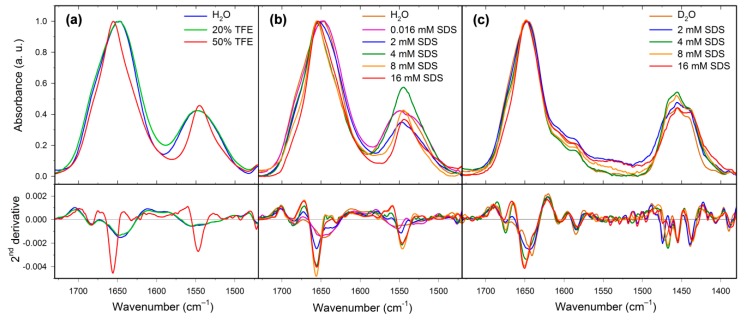
IR spectra (**top**) and their second derivatives (**bottom**) of HAL-1 (10 mg/mL) in (**a**) the presence of TFE (0%, 20%, and 50%); (**b**) of SDS/H_2_O solution (0.016, 2, 4, 8, and 16 mM SDS). (**c**) of SDS/D_2_O solution (2, 4, 8, and 16 mM SDS).

IR spectroscopy is sensitive to the β-sheet and β-turn structure and its combination with ECD may provide further conformational details. IR experiments can be carried out in aqueous solution (H_2_O or D_2_O), and the structure assignment is based on band positions within the amide I region (1600−1700 cm^−1^). For the peptides measured in H_2_O, it is usually difficult to distinguish between the α-helical and disordered structures because the corresponding amide I bands can occur in the same spectral range. This problem may be partially solved using hydrogen/deuterium exchange, which significantly reduces the α-helix and disordered structure amide I band overlaps [41]. For the natural HAL-1 measured in H_2_O, the amide I band positioned at ~1646 cm^−1^ indicates that the peptide is mainly in a random coil conformation [42] with a minor contribution of β-turns (a shoulder at ~1682 cm^−1^) [43]. Such assignment is confirmed by the IR measurements in D_2_O, where a band at ~1641 cm^−1^ (Figure 6, Table A4) can be again assigned to the random coil structure and bands at ~1658 and 1675 cm^−1^ to β-turns [41]. Upon addition of TFE (10–50% *v*/*v*), IR spectra of HAL-1 exhibit a blue shift of the amide I band from ~1646 to ~1656 cm^−1^ (Figure 6, Table A4), suggesting a secondary structure change from the random coil to the α-helical structure [42,43]. An additional band at ~1630 cm^−1^ suggests an occurrence of a β-sheet structure, and a band at ~1621 cm^−1^ indicates formation of intermolecular hydrogen bonds, typical for peptide aggregation [42,44]. The band at ~1683 cm^−1^, due to the β-turn structure, remains at the same position. A similar spectral shift of the band at ~1645 cm^−1^ to 1657 cm^−1^ indicating a conformational change from the random coil to the α-helical structure is observed in the IR spectra of HAL-1 interacting with SDS (2–8 mM). In 8 mM SDS, the presence of a shoulder at ~1686 cm^−1^ together with a spectral band at ~1631 cm^−1^ suggests formation of the β-sheet structure [23,41]. Spectral shift of the band at ~1646 to 1657 cm^−1^ can be interpreted in terms of a formation of the α-helical structure. This assumption is confirmed by an analogous measurement of the same concentration dependence in D_2_O (Figure 6, Table A4) where the main spectral component shifts from 1641 cm^−1^ (a disordered structure) in D_2_O to ~1650 cm^−1^ (an α-helical structure [42]) in SDS at a concentration above cmc (8 mM). The high-frequency component at ~1682 cm^−1^ downshifts to 1676 cm^−1^, most probably due to a β-sheet structure formation [42]. These findings are in agreement with the results of ECD analysis, showing that, besides the α-helical structure, the β-sheet structure is also present.

**Figure 7:**
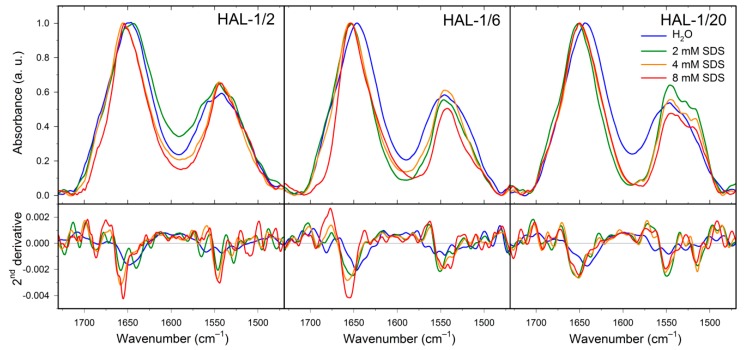
IR spectra (**top**) and their second derivatives (**bottom**) of HAL-1/2, HAL-1/6 and HAL-1/20 (10 mg/mL) in the presence of SDS (0, 2, 4, and 8 mM).

IR spectrum of HAL-1/2 in H_2_O is dominated by a band at ~1648 cm^−1^ due to the presence of a random coil structure. An additional band at ~1684 cm^−1^ and a shoulder at ~1636 cm^−1^ indicate a minor portion of β-turn and β-sheet conformation [42]. Upon addition of SDS, the band at 1648 cm^−1^ diminishes and a dominant component at ~1655 cm^−1^ shows prevailing α-helical structures. Addition of SDS again seems to cause a change in the β-structure arrangement, as in 8 mM SDS, there are only two corresponding bands at ~1686 and 1639 cm^−1^. Formation of a low-frequency band at ~1623 cm^−1^ indicates a partial peptide aggregation. Similar to HAL-1, IR spectrum of HAL-1/6 in water (Figure 7) is dominated by an amide I band at ~1646 cm^−1^ indicating prevailing random coil structure, and a lower-intensity component at ~1682 cm^−1^ assigned to β-turns [42]. Upon addition of SDS, the main amide I component shifts to ~1655 cm^−1^ (in 8 mM SDS), again hinting at a transition from a random coil to an α-helical structure. This process is accompanied by diminishing of the β-turn band at ~1682 cm^−1^ and formation of a band at ~1694 cm^−1^, implying a conformational change from the β-turn to β-sheet conformation [42]. IR spectrum of HAL-1/10 (Figure 7, Table A4) in H_2_O has the main feature at ~1643 cm^−1^ which can be assigned either to the random coil, or the β-sheet structure [41,42]. As for HAL-1, a lower-intensity band at ~1681 cm^−1^ suggests the presence of β-turns [42]. Conformational behavior of HAL-1/10 upon addition of SDS is practically the same as for HAL-1/6 (not shown). While the IR spectrum of HAL-1/20 (Figure 7, Table A4) in H_2_O is (similar to HAL-1/10) dominated by a band at ~1642 cm^−1^ due to either random coil, or β-sheet structure [41,42], a shoulder at ~1656 cm^−1^ indicates, in this case, a minor contribution of the α-helical structure [42]. Upon addition of SDS, the band at ~1642 cm^−1^ diminishes and a new band at ~1651 cm^−1^ is formed, indicating a conformational change to α-helical and/or random coil structure. The higher-frequency component, which shifts to ~1659 cm^−1^, can be assigned to a 3_10_-helix [45]. In addition, we observe a new band at ~1692 cm^−1^, reflecting the β-sheet structure formation [42].

**Figure 8:**
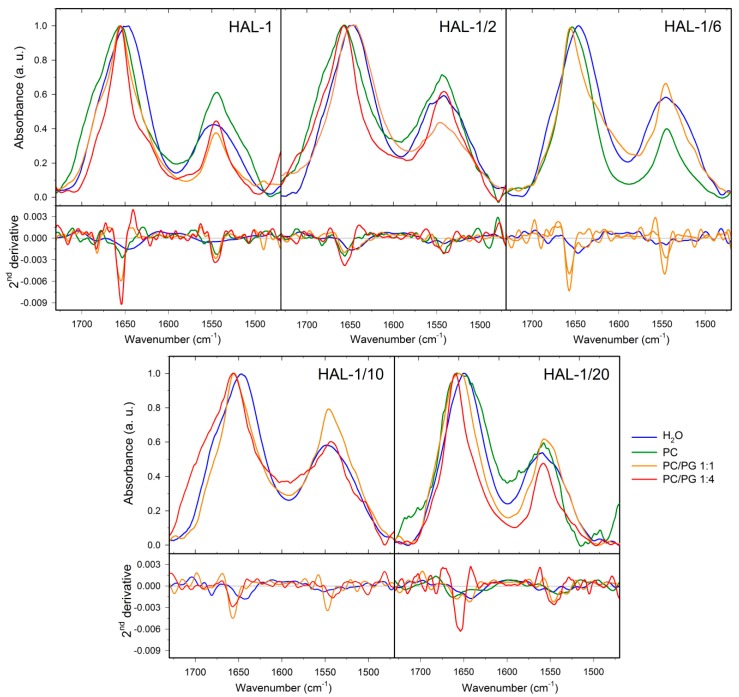
IR spectra (**top**) and their second derivatives (**bottom**) of HAL-1 and its analogs (10 mg/mL) in the presence of LUVs having different composition: in aqueous solution and in the presence of LUVs prepared from PC and PC/PG mixtures: 1:1 and 1:4.

We have already shown that the secondary structure of HAL-1 in interaction with LUVs depends on LUV composition [23]. In the presence of neutral PC-based liposomes (a model of a mammalian membrane [1]), the dominant amide I band shifts to ~1652 cm^−1^ probably due to the simultaneous presence of a random coil and an α-helical structure. Bands at ~1686 and 1619 cm^−1^ relate to the formation of β-turns and β-sheet aggregates (Table A3). With an increasing fraction of negatively charged PG in the liposomes, the β-aggregates (the band at ~1619 cm^−1^) almost disappear and the α-helical structure content increases (formation of a band at ~1655 cm^−1^) with a continuous diminishing of the β-turn content (the band at ~1686 cm^−1^). IR spectra of HAL-1/2 (Figure 8, Table A4) display a notable spectral shift of the amide I band (from ~1648 to 1656 cm^−1^) already in the presence of neutral PC-based liposomes indicating a conformational change from an unordered to the α-helical structure. The highest α-helical fraction is observed for HAL-1/2 interacting with negatively charged liposomes composed of PC/PG in a 1:4 ratio. An increase in α-helical structure content is accompanied by diminishing of the β-sheet bands (at ~1640 and 1690 cm^−1^) and also some minor changes in the β-turn arrangement (a slight shift of the band at ~1685 cm^−1^ to 1681 cm^−1^). Similar to HAL-1/2, the formation of a band at ~1657 cm^−1^, indicating an increase in the α-helical structure content, is observed also for HAL-1/6 upon addition of neutral PC-based liposomes (Figure 8). An additional band at ~1639 cm^−1^ is probably due to the β-sheet structure formation.

For HAL-1/10 in the presence of LUVs, with an increasing fraction of PG in the liposomes, we observe diminishing of the band at ~1643 cm^−1^ and formation of a band at ~1656 cm^−1^, indicating again a transition from β-sheet and/or random coil structure to α-helical structure. This process is accompanied by diminishing of the band at ~1681 cm^−1^ assigned to β-turns and formation of a band at ~1690 cm^−1^ probably due to β-sheet structure occurrence [41,42] (Figure 8, Table A4). FTIR spectra of HAL-1/20 in the presence of neutral PC-based liposomes indicate the formation of β-sheet aggregates (band at ~1626 cm^−1^). A dominant spectral component at ~1663 cm^−1^ can be probably assigned to the 3_10_-helical structure. These two spectral features disappear with an increasing fraction of PG in the liposomes and we observe formation of a band due to the α-helical structure (positioned at ~1658 and 1654 cm^−1^ in the presence of liposomes composed of PC/PG in the 1:1 and 1:4 ratio, respectively). For this analog, bands assigned to the β-sheet (at ~1634 and 1694 cm^−1^) and β-turn structures (at ~1681 cm^−1^) can be observed even in the presence of liposomes with the highest PG fraction (PC/PG in the 1:4 ratio).

IR spectroscopy confirms induced conformational change from the random coil to the α-helical structure in the biologically active HAL-1 analogs upon interaction with the bacterial membrane models and complements ECD results by providing information about the β-structure formation. Based on the results of IR analysis, spectral changes in ECD curves observed for the peptides interacting with SDS in concentration far above cmc and for PG-containing LUVs are probably due to changes in the β-sheet content. Behavior of HAL-1 peptides towards LUVs reveals (a) in accordance with biological data, the most active natural HAL-1 shows the lowest tendency to form β-aggregates and the β-sheet structure upon interaction with PG-containing LUVs; (b) contrary to the ECD results, all HAL-1 analogs adopt some portion of the α-helical structure already in the presence of neutral liposomes (representing a crude model of mammalian cells, against which the peptides have no or very low activity). This rather surprising conformational behavior could be caused by different experimental conditions used for the measurements of IR and ECD spectra (IR experiments require ~100× higher peptide concentration than ECD measurements, see experimental conditions for ECD and IR measurements in the Materials and Methods section). It seems that under such conditions, the peptides tend to form specific assemblies, adopting a conformation with a high α-helical content.

### 2.3. Concentration Dependence Measurements

**Figure 9:**
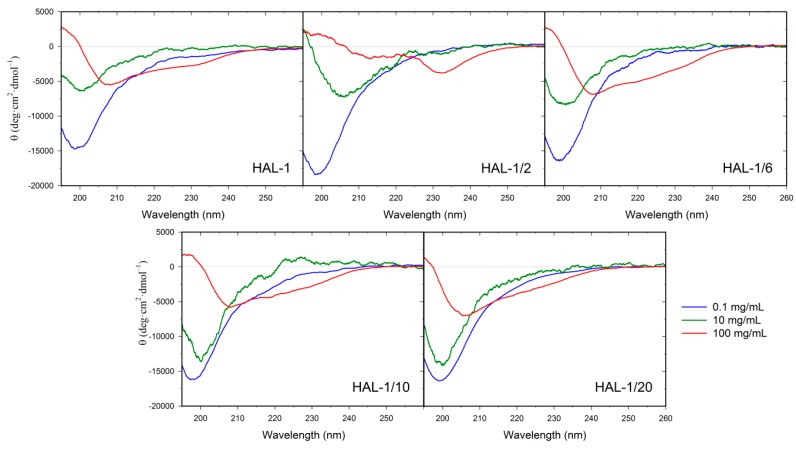
Concentration dependence of ECD spectra of HAL-1 and its analogs: 0.1, 10, 100 mg/mL.

**Figure 10:**
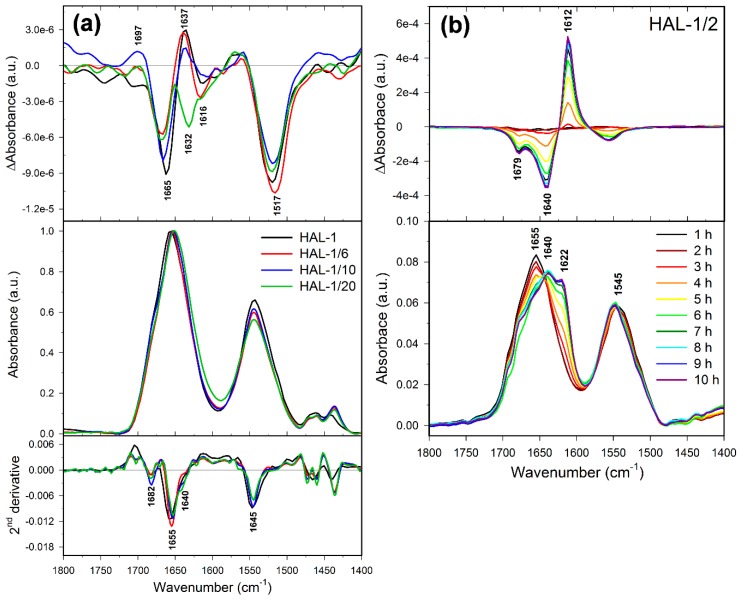
(**a**) VCD (**top**)/IR (**middle**)/second derivatives of IR spectra (**bottom**) of HAL-1, HAL-1/6, HAL-1/10, and HAL-1/20 in aqueous solution for samples in the 100 mg/mL concentration; (**b**) time dependence (1–10 h after preparation) of VCD (**top**) and IR spectra (**bottom**) of HAL-1/2 in aqueous solution for the sample in the 100 mg/mL concentration.

In order to clarify the discrepancy between the results of the peptide conformational analyses obtained by ECD and IR, we have complemented our study by a measurement of concentration dependencies of ECD spectra of the HAL-1 peptides (Figure 9). Our ECD data indicate that with increasing peptide concentration, the unordered/PPII conformation changes to a partially α-helical conformation for all the studied analogs except for HAL-1/2, which seems to form β-aggregates when its concentration reaches 100 mg/mL. In order to determine the peptide conformational change induced by its high concentration more specifically, we have performed measurements of VCD spectra of the peptides (Figure 10) using the highest peptide concentration studied by ECD (i.e., 100 mg/mL). VCD measurements supported the results of ECD analysis, confirming that at high peptide concentration, HAL-1 and its analogs HAL-1/6, HAL-1/10, and HAL-1/20 spontaneously form the α-helical structure, indicated by a negative/positive VCD couplet in the amide I region [46,47,48]. On the contrary, the HAL-1/2 analog (Figure 10b) seems to undergo a distinct conformational change, forming highly organized β-sheet aggregates (β-sheet fibrils) characterized by an intense five-peak VCD signal with the (−/−/+/−/−) sign pattern in the amide I and II region [49,50,51]. Interestingly, a tendency to form the α-helical structure at high peptide concentration is common for all the HAL-1 analogs except for HAL-1/2, which exhibits the most reduced biological activity. It is therefore possible that for the peptide biological action, formation of specific assemblies with a high α-helical content might be also important.

### 2.4. Structural Changes Followed by Fluorescence Spectroscopy

Participation of the tryptophan residue in the interaction of HAL-1 peptides with SDS and LUVs can be monitored selectively on the basis of fluorescence spectra. The observed fluorescence signals are assigned to the Trp3 residue. In aqueous solutions, the peptides exhibit fluorescence maxima at 360 nm (HAL-1), 356 nm (HAL-1/2), 361 nm (HAL-1/6) and 359 nm (HAL-1/20) (Table 2), i.e., the typical values for Trp residue in a hydrophilic environment. Upon interaction with LUVs, these maxima shift to about 330 nm (Table 2) indicating that tryptophan is not fully immersed in the lipophilic part of the liposome, but it is still in a close proximity of liposome phosphate heads [52]. In the case of HAL-1/2, the fluorescence maximum shifts only to 350 nm. Hence, it is probable that HAL-1/2 while interacting with LUVs, does not incorporate itself into the liposome despite the fact that parallel ECD experiments indicate a formation of some α-helical secondary structure. This result confirms our assumption that the α-helix formation is an important but not a sufficient condition for the efficient functioning of our AMPs. This conclusion is further supported by the results shown by HAL-1/6. Although HAL-1/6 readily interacts with LUVs by forming the α-helical structure, its biological activity is smaller than the activity of the natural HAL-1. Fluorescence spectroscopy shows that HAL-1 peptides are attached to the membrane surface with little penetration, indicating that the HAL-1 peptide mode of action probably involves either (a) dissolving the membrane in a detergent-like manner (the carpet model) or (b) formation of toroidal-type trans-membrane pores (lined both by peptide molecules and phospholipid headgroups) [53].

### 2.5. Molecular Dynamics

**Figure 11:**
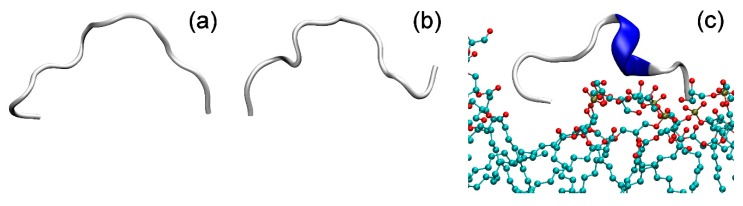
Molecular mechanic simulation of HAL-1 peptide (**a**) in water, (**b**) in the presence of PC and (**c**) PG model membrane.

As follows from the fluorescence spectroscopy measurements, HAL-1 peptides seem to bind to the outer leaflet of our model membranes. In order to better understand the mechanism of peptide-membrane interaction, we performed MD simulations of HAL-1 in water and in the presence of PC and PG-based model membranes. According to our results, HAL-1 in water is in a random coil conformation (Figure 11). When in the vicinity of a PC containing membrane, HAL-1 does not immerse into the membrane and it seems to have no defined orientation with regards to the membrane. HAL-1 does not change its structure and it still adopts a random coil conformation. The result of this simulation might seem rather surprising: Although the peptide exhibits hemolytic activity, we do not observe any peptide-membrane interaction. However, as follows from our spectroscopic results (see Section 2.3), HAL-1 structural behavior is concentration dependent and it is therefore probable that for its full activity, it is necessary to exceed a certain peptide threshold concentration. Under such conditions, the peptide might form specific assemblies (e.g., pores) that would allow for the peptide-membrane interaction [54].

When put into interaction with the PG based membrane, HAL-1 is anchored to the membrane by the terminal amino acid Arg. The peptide does not penetrate into the membrane and for the simulation time (110 ns) it stays in the vicinity of the membrane (Figure 11), which is in agreement with the fluorescence spectroscopy results, showing that tryptophan is in close proximity to the liposome phosphate heads (see Section 2.4). Based on the MD simulations, HAL-1 in a bacterial membrane-mimicking environment adopts mostly a 3_10_-helical structure with a minor portion of the β-turn structure (Figure 11). Such a result does not contradict the ECD and IR data (suggesting under such conditions, formation of the α-helical conformation), as it was shown that the 3_10_-helix is an important intermediate along the α-helix folding/unfolding pathway [55].

Based on the results of our spectroscopic investigation and MD simulations, we suggest a mechanism of HAL-1 interaction with model membranes: HAL-1 as a cationic peptide is mostly attracted to the negatively charged leaflets of the PG based model membranes, adopting predominantly a 3_10_-helical conformation—an intermediate conformation and/or a precursor of the α-helical structure [55]. Since the peptide in such conformation already adopts an amphipathic structure, formation of peptide assemblies (most probably membrane pores), where the peptides are predominantly in the α-helical conformation, seems favorable. However, for the formation of such assemblies, it is necessary to exceed a specific threshold concentration of the peptide in a close proximity or in an immediate contact with the membrane. The proposed mechanisms of action are inspired by the molecular mechanisms of cooperativity of antibacterial peptides proposed by Huang et al. [54,56], and correspond to the mechanism and dynamics of AMP channel formation monitored *in situ*, where at least a three-step procedure of AMP insertion was suggested and the importance of the peptide–peptide interaction was demonstrated [57]. Several steps need to be taken to confirm this hypothesis: (1) It would be beneficial to perform more profound MD simulations of all HAL-1 analogs interacting with membranes of different composition. A peptide concentration dependency should be studied, investigating two or more peptides that interact on a membrane surface; (2) this computational study should be complemented by experiments allowing detailed study of peptide orientation with respect to the membrane such as oriented ECD [54], surface-enhanced infrared absorption spectroscopy [57], or oriented solid-state NMR measurements [58]. This will be a matter of further investigation.

## 3. Materials and Methods

### 3.1. Materials

The phospholipids, PC, and PG (sodium salt), were purchased from Avanti Polar Lipids (Alabaster, AL, USA). TFE was purchased from Merck (Darmstadt, Germany) and SDS from Sigma (Darmstadt, Germany). The peptides (see Table 1) were prepared by the standard procedures of solid phase peptide synthesis [19]. All the peptides were delivered as TFA salts (with TFA counterions bonded to the free amino termini and side chains of positively charged amino acids). For the natural HAL-1, it was possible to remove TFA counterions using a standard procedure [59] as there was a sufficient amount of the sample available.

### 3.2. Preparation of Vesicles

The phospholipids PC, PG or their mixtures (at 1:4, 1:1 or 4:1 molar ratios) were dissolved in chloroform/methanol (3:1) mixture and dried under vacuum. The dry lipid layer was hydrated with distilled water and gently stirred. LUVs with an approximate diameter of 0.1 µm were formed by extrusion through polycarbonate membranes (pore size 0.1 µm, a total of 30 passages through the membrane) using Mini-Extruder (Avanti Polar Lipids, Alabaster, AL, USA). The temperature of the lipid suspension was kept above the phase transition temperature *T*_m_ of the lipid with the highest *T*_m_ within the whole hydration and extrusion process. A liposome size of 0.1 µm was selected in order to avoid artifacts due to light scattering (especially in ECD experiments). The shapes and sizes of liposomes were checked by cryo-electron microscopy. Liposome stabilities and size distributions were verified by light scattering using Zetasizer Nano (Malvern Panalytical, Malvern, UK).

### 3.3. Electronic Circular Dichroism

ECD experiments were carried out on J-815 spectropolarimeter (Jasco, Tsukuba, Japan) equipped with the Peltier type temperature control system PTC-423S/L. The spectra were collected from 180 to 300 nm at room temperature in 0.1 cm quartz cells (0.125 mg/mL peptide concentration, 2 scans, 0.5 nm steps, 20 nm/min speed, 8 s time constant, 1 nm spectral bandwidth). For the measurements in high liposome concentration (L/P concentration ratio higher than 100), the cell with 0.02 cm path length and appropriate experimental conditions (5 nm/min speed, 32 s response time and 1 nm bandwidth) were used. After baseline subtraction, the final data were expressed as molar ellipticities θ (deg·cm^2^·dmol^−1^) per residue. All samples were prepared by dilutions of a stock peptide solution (1 mg/mL) to a final peptide concentration 0.125 mg/mL, followed by adding an appropriate aliquot of TFE (final TFE concentration 10–50% *v*/*v*), SDS (stock solution 32 mM, final SDS concentration 0.016–16 mM, i.e., below and above cmc; cmc ≈ 4 mM for the SDS-peptides solution [60]), or LUVs (stock solution 200 mg/mL). SDS measurements below and above cmc enable investigating the effects of SDS acting both as (a) a denaturation agent (below cmc) and (b) a simple membrane model (above cmc).

Concentration dependence was measured for all the peptides at the given concentrations: 0.1, 10, and 100 mg/mL. The spectra were collected from 180 to 300 nm at room temperature in 0.1 cm quartz cells for the 0.1 mg/mL peptide concentration, and in 6 µm homemade CaF_2_ cells for 10 mg/mL and 100 mg/mL peptide concentration with the following setup: 2 scans, 0.5 nm steps, 20 nm/min speed, 8 s time constant, 1 nm spectral bandwidth. The α-helical fraction was calculated using a two-state model [30,61]. For the more detailed analysis of secondary structure, we used the CDPro software package [36,62].

### 3.4. Principle Component Analysis

Analysis of temperature- and time-dependent ECD spectra was performed using principal component analysis (PCA) based on a singular value decomposition algorithm applied to reduce spectral series {*Y*_i_(ν), *i* = 1, …, *n*} to their lowest dimension without the loss of spectroscopic information. Each spectrum of the matrix *Y*_i_(ν) can be unambiguously expressed as:

(1) $$Y_{i}(\nu )=\sum_{j=1}^{M}V_{ij}W_{j}S_{j}(\nu )$$

where *W_j_* is the diagonal matrix of singular values, *S*(*ν*) corresponds to the matrix of the orthonormal subspectra (eigenvectors) and *V_ij_* is the unitary square matrix of coefficients (representing the influence strength of the subspectrum *S_j_*). *M* represents a number of independent “spectral species”, distinct from the spectral noise, found in the analyzed data set. The number of independent subspectra can be estimated from residual errors or from singular values. A detailed explanation of PCA can be found in [63]. The calculation of PCA was done using our own software programmed in Matlab™ (MathWorks^®^, Natick, MA, USA).

### 3.5. Infrared Spectroscopy

IR spectra in the transmission mode were recorded on Nicolet 6700 spectrometer (Thermo Fisher Scientific, Waltham, MA, USA) using standard mid-IR source, KBr beamsplitter and DTGS detector (2 cm^−1^ spectral resolution, Happ–Genzel apodization function, 2000 scans) in the 4000–1000 cm^−1^ spectral range. The cell compartment was purged by dry nitrogen during all the measurements. Aqueous solutions (10 mg/mL peptide concentration) were measured at room temperature in homemade CaF_2_ cells with 6 µm path length (cell volume 1 µL). In our experiments involving peptide-membrane interaction, the L/P was always equal to 8 (LUVs stock solution 200 mg/mL). Numerical data treatment was carried out using Grams/AI software (Thermo Electron, Waltham, MA, USA). The spectral contribution of water was eliminated using a standard algorithm [64]. The IR signal of phospholipids was subtracted from spectra of peptide/phospholipid mixtures. Subsequently, the spectrum of water vapors was subtracted and the baseline was linearly corrected. Final IR spectra were normalized to amide I intensity maxima. The IR spectra of HAL-1 analogs were obtained by additional subtraction of trifluoroacetate signals, which were present due to a standard cleavage from the resin by TFA [65]. Such subtraction was not needed for the natural HAL-1 as for this peptide, the TFA counterions were successfully removed (see Section 3.1). The secondary structure analysis [41] was aided using second derivatives Savitzky−Golay algorithm (Grams/AI software, Thermo Electron, Waltham, MA, USA) and a band fitting procedure (Gaussian−Lorentzian band shape—OMNIC Thermo Fisher Scientific, Waltham, MA, USA).

### 3.6. Vibrational Circular Dichroism

VCD spectra were measured on a dual source [66] and dual photo-elastic modulator [67] VCD spectrometer ChiralIR-2X™ (BioTools, Jupiter, FL, USA) at room temperature in CaF_2_–BioCell™ with 6 µm path length (BioTools, Jupiter, FL, USA). The data were collected for ~12 h (12 blocks of 6000 scans each at 8 cm^−1^ resolution). The spectra were processed in Grams/AI software (Thermo Electron, Waltham, MA, USA). Solvent scans were subtracted as background. Baseline was corrected using a linear function. The spectra were smoothed with a second-order Savitzky-Golay filter using a 9 point window and normalized to amide I maxima in the corresponding IR spectra.

### 3.7. Fluorescence Spectroscopy

Steady-state fluorescence spectra were measured on Fluoromax Z (Jobin-Yvon, Chilly-Mazarin, France) fluorimeter in a 10 mm quartz cell. Excitation at 280 nm was used to induce fluorescence of the tryptophan residue. The emission was collected from 300 to 450 nm with the 1.5 s integration time. The emission and excitation slits were chosen as 2 nm. The peptide concentration of 0.125 mg/mL was chosen identical as for the ECD experiments in order to maintain mutual compatibility. At this low concentration, there is no danger of Trp residues self-quenching. L/P = 20 was used for all the fluorescence measurements (2 mg/mL lipid concentration).

### 3.8. Molecular Dynamics

An all-atom structure model of the peptides was created using the tLEaP program from the AmberTools (San Francisco, CA, USA) [68] suite and the force field Amber FF99SB [69] PC and PG membrane models were created using program VMD [70] and its membrane plugin. PC and PG parameters were generated using programs Antechamber and Parmchk from the AmberTools suite. In total, three systems were created, a peptide in water and the peptide with PC and PG membranes. Each system was solvated in TIP3P water and neutralized using K^+^ and Cl^−^ ions. The initial equilibration of systems was performed using NAMD 2.9 [71] with a time step of 1 fs and rigid bonds in water molecules using Settle algorithm [72]. Systems were minimized for 1000 steps, warmed to 310 K and equilibrated for 1 ps. The system without a membrane was simulated for 10 ps, and the systems with membranes were simulated for 110 ps.

## 4. Conclusions

The combined use of the methods of molecular spectroscopy (ECD, IR absorption, VCD and fluorescence spectroscopy) together with MD simulations allowed us to follow secondary structure changes of HAL-1 and its analogs induced by an interaction with artificial membrane models. On the basis of the obtained results, formation of the α-helical structure appears important for the activity of the HAL-1 peptides. However, peptide biological activities seem to be determined not only by their propensity to form the α-helical structure. Additional factors like the ability of the peptides to adopt alternative conformations (such as β-sheet, PPII conformation or 3_10_-helix) cannot be excluded and have to be considered for their biological activity as well. For biologically active analogs, the concentration-dependence ECD measurements together with VCD data suggest a possible formation of peptide assemblies high in α-helical content (most probably membrane pores), which might enable membrane penetration. Following our spectroscopic results, we can propose that HAL-1 structural behavior is concentration dependent and for its full activity, certain peptide threshold concentration should be exceeded. Under such conditions, the peptides might form specific assemblies (e.g., pores) that would allow for the peptide-membrane interaction and complete their task as antimicrobial agents.

## Figures and Tables

**Table 1:** Amino acid sequences, physico-chemical and biological properties (µ_H_ is the hydrophobic moment, and *H* represents the mean hydrophobicity, calculated according to [27]), of the studied antibacterial HAL-1 peptides. Data were taken from ref. [19]. Point mutations with respect to the natural HAL-1 peptide are underlined. The Schiffer−Edmundson wheel projection of the HAL-1 and its analogs is depicted below the table.

Acronym	Sequence	MW (Da)	Charge	µ_H_	*H*	Antimicrobial Activity MIC (µM)				Hemolytic LC_50_ (µM)
						B.^1^	S.^2^	E.^3^	P.^4^	
HAL-1	GMWSKILGHLIR	1408.9	+3	0.380	−0.004	0.8	7.7	3.8	45.0	82
HAL-1/2	GMWSKILGPLIR	1368.8	+3	0.361	+0.023	3.6	>100	30.0	>100	>200
HAL-1/6	GMWSKILGHLIK	1380.6	+3	0.323	+0.051	1.3	15.8	7.2	65.0	132
HAL-1/10	GMWKKILGKLIR	1440.9	+5	0.416	–0.133	0.8	15.0	2.3	13.1	>200
HAL-1/20	GKWSKILGKLIR	1396.9	+5	0.473	–0.176	1.7	21.7	2.3	28.3.	>200
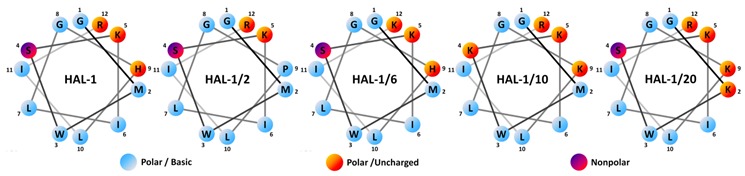										

^1^*Bacillus subtilis*, ^2^*Staphylococcus aureus*, ^3^*Escherichia coli*, ^4^*Pseudomonas aeruginosa*.

**Table 2:** Tryptophan fluorescence maxima of HAL-1 and its analogs (0.125 mg/mL—identical as for the ECD experiments) in aqueous solution and in the presence of LUVs (L/P = 20).

Solution	HAL-1	HAL-1/2	HAL-1/6	HAL-1/20
Water	360 nm	356 nm	361 nm	359 nm
PC	356 nm	362 nm	356 nm	359 nm
PC/PG (1:1)	331 nm	350 nm	336 nm	333 nm

## Abbreviations

AMPs	Antimicrobial peptides
cmc	Critical micelle concentration
ECD	Electronic circular dichroism
HAL	Halictine
IR	Infrared
L/P	Lipid/peptide ratio
LUV	Large unilamellar vesicle
MD	Molecular dynamics
PC	1,2-Dimyristoyl-*sn*-glycerol-3-phosphatidylcholine
PCA	Principal component analysis
PG	1,2-Dimyristoyl-*sn*-glycero-3-phospho-(1′-*rac*-glycerol)
PPII	Polyproline II
SDS	Sodium dodecyl sulfate
TFE	2,2,2-Trifluoroethanol
VCD	Vibrational circular dichroism

## Appendix A

**Table A1:** Helical fraction for HAL-1 and its analogs in aqueous solution and in the presence of TFE (concentration expressed in volume percent (*v*/*v*)) and SDS (in mM) calculated using a two-state model from ECD spectra [30,61].

Solution	Hal-1	Hal-1/2	Hal-1/6	Hal-1/10	Hal-1/20
Water	14%	14%	13%	15%	15%
TFE 30%	32%	32%	37%	36%	36%
TFE 50%	32%	32%	40%	42%	36%
SDS 0.016 mM	13%	13%	3%	14%	14%
SDS 0.16 mM	14%	14%	3%	18%	52%
SDS 2 mM	46%	46%	66%	63%	36%
SDS 4 mM	51%	51%	46%	63%	32%
SDS 8 mM	37%	37%	46%	50%	31%
SDS 16 mM	31%	31%	36%	36%	42%

**Table A2:** Estimation of the secondary structure content calculated from ECD spectra using the CDPro package [62,73] for HAL-1 and its analogs in aqueous solution, and in the presence of SDS.

Structure	Sodium Dodecyl Sulfate						
	0 mM	0.016 mM	0.16 mM	2 mM	4 mM	8 mM	16 mM
**HAL-1**							
α-helix	12%	12%	14%	61%	61%	53%	53%
β-sheet	50%	50%	44%	6%	6%	10%	10%
β-turn	16%	16%	16%	12%	12%	17%	17%
Other	22%	22%	26%	22%	22%	21%	21%
**HAL-1/2**							
α-helix	12%	11%	14%	14%	47%	47%	44%
β-sheet	53%	54%	45%	44%	13%	13%	15%
β-turn	16%	16%	16%	16%	18%	18%	18%
Other	19%	19%	25%	25%	22%	22%	24%
**HAL-1/6**							
α-helix	12%	13%	11%	82%	85%	70%	60%
β-sheet	52%	42%	37%	2%	2%	4%	9%
β-turn	16%	16%	14%	9%	9%	14%	19%
Other	20%	29%	38%	6%	4%	11%	14%
**HAL-1/10**							
α-helix	11%	12%	13%	80%	79%	72%	70%
β-sheet	52%	51%	43%	2%	2%	4%	5%
β-turn	16%	16%	16%	10%	10%	14%	15%
Other	21%	21%	27%	8%	9%	10%	10%
**HAL-1/20**							
α-helix	12%	13%	66%	42%	39%	37%	60%
β-sheet	52%	42%	5%	13%	17%	18%	18%
β-turn	16%	13%	12%	17%	18%	18%	17%
Other	20%	32%	18%	28%	27%	27%	15%

**Table A3:** Estimation of the secondary structure content calculated from ECD spectra using the CDPro package [62,73] for HAL-1 and its analogs in aqueous solution, and in the presence of LUVs.

Structure	LUV					
	0 mM	PC (L/P = 20)	PC (L/P = 100)	PC/PG (4:1) (L/P = 20)	PC/PG (1:1) (L/P = 20)	PC/PG (1:4) (L/P = 20)
**HAL-1**						
α-helix	12%	12%	18%	35%	45%	61%
β-sheet	50%	51%	38%	17%	10%	7%
β-turn	16%	16%	18%	18%	17%	12%
Other	22%	21%	26%	30%	22%	21%
**HAL-1/2**						
α-helix	11%	11%	17%	37%	47%	69%
β-sheet	53%	53%	40%	17%	10%	5%
β-turn	16%	16%	18%	17%	16%	14%
Other	19%	21%	25%	29%	28%	13%
**HAL-1/6**						
α-helix	12%	12%	14%	60%	79%	60%
β-sheet	52%	50%	45%	7%	1%	6%
β-turn	16%	16%	17%	8%	5%	12%
Other	20%	22%	24%	25%	14%	21%
**HAL-1/10**						
α-helix	11%	12%	13%	35%	69%	36%
β-sheet	52%	52%	51%	18%	3%	38%
β-turn	16%	16%	17%	17%	9%	14%
Other	21%	20%	19%	30%	7%	13%
**HAL-1/20**						
α-helix	12%	12%	14%	25%	80%	46%
β-sheet	52%	50%	46%	24%	2%	13%
β-turn	16%	16%	17%	18%	10%	18%
Other	20%	21%	22%	34%	7%	24%

**Table A4:** Estimation of the secondary structure content from IR spectra, using a bandfitting procedure for HAL-1 and its analogs in aqueous solution (H_2_O and D_2_O), in the presence of SDS, TFE, and LUVs. The numbers in parentheses correspond to percentages of each fitted band in the amide I region.

Solvent	Amide I (cm^−1^)	Second Derivative Decomposition of Amide I (cm^−1^)						
		aggregate	β-sheet	α-helix	3_10_-helix	coil	β-turn	β-sheet
**HAL-1**								
H_2_O	1646	—	—	—	—	1648 (88)	1683 (12)	—
D_2_O	1647	—	—	—	—	1642(69)	1667 (31)	—
TFE	1655	—	1633 (26)	1656 (63)	—	—	1680 (11)	—
SDS 8 mM/H_2_O	1655	1621 (12)	1635 (16)	1656 (63)	—	—	1682 (9)	—
SDS 8 mM/D_2_O	1649	—	—	1649 (95)	—	—	1676 (5)	—
PC	1656	1619 (5)	—	1656 (63)	—	—	1688 (33)	—
PC/PG 1:1	1655	—	1634 (29)	1656 (52)	—	—	1680 (19)	—
PC/PG 1:4	1656	—	1633 (6)	1655 (58)	—	—	1678 (36)	—
**HAL-1/2**								
H_2_O	1649	—	1637 (29)	—	—	1650 (56)	1684 (14)	—
SDS 8 mM/H_2_O	1655	—	1635(38)	1657 (58)	—	—	1685 (4)	—
PC	1657	—	1642 (32)	1657 (32)	—	—	1680 (35)	—
PC/PG 1:1	1647	1628 (34)	1642 (28)	1658 (24)	—	—	1676 (13)	—
PC/PG 1:4	1657	—	1632 (15)	1656 (43)	—	—	1680 (41)	—
**HAL-1/6**								
H_2_O	1646	—	—	—	—	1647 (92)	1682 (8)	—
SDS 8 mM/H_2_O	1654	1629 (29)	—	1655 (69)	—	—	—	1695 (2)
PC	1654	1621 (7)	1639 (29)	1657 (59)	—	—	1680 (5)	—
PC/PG 1:1	1656	1626 (9)	1642 (18)	1657 (62)	—	—	1682 (11)	—
**HAL-1/10**								
H_2_O	1647	—	—	—	—	1647 (82)	1681 (18)	—
PC/PG 1:1	1655	1625 (12)	1640 (21)	1657 (57)	—	—	1681 (10)	—
PC/PG 1:4	1656	—	—	1656 (80)	—	—	1688 (20)	—
**HAL-1/20**								
H_2_O	1644	—	1643 (93)	—	—	—	1676 (7)	—
SDS 8 mM/H_2_O	1650	1629 (9)	—	1653 (83)	—	—	1685 (9)	—
PC	1649	1625 (36)	1639 (13)	1654 (28)	—	—	1685 (22)	—
PC/PG 1:1	1650	1628 (29)	1642 (16)	1659 (49)	—	—	1685 (6)	—
PC/PG 1:4	1654	1621 (19)	1634 (14)	1655 (43)	—	—	1678 (24)	—
